# Classification of rib fracture types from postmortem computed tomography images using deep learning

**DOI:** 10.1007/s12024-023-00751-x

**Published:** 2023-11-16

**Authors:** Victor Ibanez, Dario Jucker, Lars C. Ebert, Sabine Franckenberg, Akos Dobay

**Affiliations:** 1 Forensic Machine Learning Technology Center, Zurich Institute of Forensic Medicine, University of Zurich, Winterthurerstrasse 190/52, CH-8057 Zurich, Switzerland; 2 Diagnostic and Interventional Radiology, University Hospital Zurich, Rämistrasse 100, 8091 Zurich, Switzerland; 3 Zurich Institute of Forensic Medicine, 3D Centre Zurich, University of Zurich, Winterthurerstrasse 190/52, CH-8057 Zurich, Switzerland

**Keywords:** Deep learning, Forensic sciences, Rib fracture classification, Postmortem computed tomography

## Abstract

Human or time resources can sometimes fall short in medical image diagnostics, and analyzing images in full detail can be a challenging task. With recent advances in artificial intelligence, an increasing number of systems have been developed to assist clinicians in their work. In this study, the objective was to train a model that can distinguish between various fracture types on different levels of hierarchical taxonomy and detect them on 2D-image representations of volumetric postmortem computed tomography (PMCT) data. We used a deep learning model based on the ResNet50 architecture that was pretrained on ImageNet data, and we used transfer learning to fine-tune it to our specific task. We trained our model to distinguish between “displaced,” “nondisplaced,” “ad latus,” “ad longitudinem cum contractione,” and “ad longitudinem cum distractione” fractures. Radiographs with no fractures were correctly predicted in 95–99% of cases. Nondisplaced fractures were correctly predicted in 80–86% of cases. Displaced fractures of the “ad latus” type were correctly predicted in 17–18% of cases. The other two displaced types of fractures, “ad longitudinem cum contractione” and “ad longitudinem cum distractione,” were correctly predicted in 70–75% and 64–75% of cases, respectively. The model achieved the best performance when the level of hierarchical taxonomy was high, while it had more difficulties when the level of hierarchical taxonomy was lower. Overall, deep learning techniques constitute a reliable solution for forensic pathologists and medical practitioners seeking to reduce workload.

## Introduction

Rib fractures are a common type of injury. They can result from blunt trauma in an accident, chest compression during cardiopulmonary resuscitation, or a pathological fracture in malignant disease. They are often associated with other injuries, such as hemo- or pneumothorax and lung contusions [1]. Depending on the displacement, type, and extent, rib fractures can result in an unstable chest (flail chest) and—in combination with associated injuries—can significantly influence morbidity and mortality [1, 2]. Depending on trauma severity or case circumstances, conventional radiography is the primary technique used to look for rib fractures because of its general availability, low radiation dose, and affordable costs. However, the sensitivity of conventional radiographs for the detection of rib fractures (especially nondisplaced ones) is considered relatively low [3, 4]. In contrast, computed tomography (CT) shows much higher sensitivity in detecting rib fractures, providing more detailed two-dimensional images that might also be viewed in three dimensions [5]. However, CT scans might not be available everywhere. In addition, they are more expensive, and they expose the patient to a higher radiation dose than conventional radiography [6]. In forensic medicine, concerns regarding radiation dose can obviously be ignored, and postmortem computed tomography (PMCT) has already gained great acceptance worldwide as a valuable adjunct and sometimes even a replacement for conventional autopsies [7].

Several recent studies have employed deep learning and image processing to automate rib fracture detection, adding to previous literature in which different groups proposed solutions for automating the detection of rib fractures on CT scans and radiographs [8–12]. For example, one recent study focused on detecting rib fractures on CT scans and classifying them into six categories, including displaced versus nondisplaced, buckle, and segmental fractures [13]. The authors trained a U-Net-based network using the RibFrac challenge dataset [14]. The model proposed by Choi et al. can also determine the position of a fracture. In another study by Wang and Wang, the authors developed a modified U-Net architecture, combined with an attention module and a modified dilated convolution, to detect and segment rib fractures on CT scans [15]. The authors relied on the same RibFrac challenge dataset to train their architecture. In a third study, Wu et al. utilized chest radiographs and employed a YOLOv3-based convolutional neural network (CNN) for rib fracture detection [16].

In our study, we developed a model to automatically detect rib fractures and classify whether they are displaced or nondisplaced using two-dimensional planar views of the rib cage reconstructed from PMCT volumetric data.

## Materials and methods

### Ethics

The data used in this retrospective cohort study are in accordance with Swiss laws and ethical standards. The ethics approval for this study was waived by the Ethics Committee of the Canton of Zurich (KEK ZH-No. 15–0686).

### Case selection

A total of 340 consecutive autopsy cases were retrospectively retrieved from July 2017 to April 2018 from the archives of the Institute of Forensic Medicine, University of Zurich, Switzerland. We excluded cases with signs of advanced decomposition (using the RA-index defined by Egger et al. [17]), corpses that had undergone organ explantation, cases of severe trauma with extensive damage to the corpse (e.g., amputation or exenteration), cases without whole-body PMCT, cases where rib fractures were not visible in the rib unfolding tool or located in the cartilaginous part of the rib, and cases that were still under investigation during this period. After these exclusion criteria were applied, a total of 195 cases remained (55 females, median age 64 years; 140 males, median age 54 years). Of the 195 cases, 85 showed acute rib fractures, 84 had no rib fractures, and 26 presented subacute and chronic fractures either in combination with acute fractures or independently. Both complete and incomplete rib fractures were included, independent of their location. They were classified as either “displaced,” “nondisplaced,” “ad latus” (sideways), “ad axim” (with angulation), “ad longitudinem cum contractione” (in long axis compressed fracture), and “ad longitudinem cum distractione” (in long axis with gap between the fragments) fractures.

### Postmortem computed tomography data

Whole-body imaging was performed on a 128-slice dual source CT scanner (SOMATOM Flash Definition, Siemens, Forchheim, Germany) using automated dose modulation software (CARE Dose4D™, Siemens, Forchheim, Germany); the slice thickness was 1 mm, and the increment was 0.5 mm. The images were reconstructed with both soft and hard kernels. A complete overview of the technical parameters used to acquire the CT scans can be found in Flach et al. [18].

### Image treatment prior to classification

**Fig. 1:**
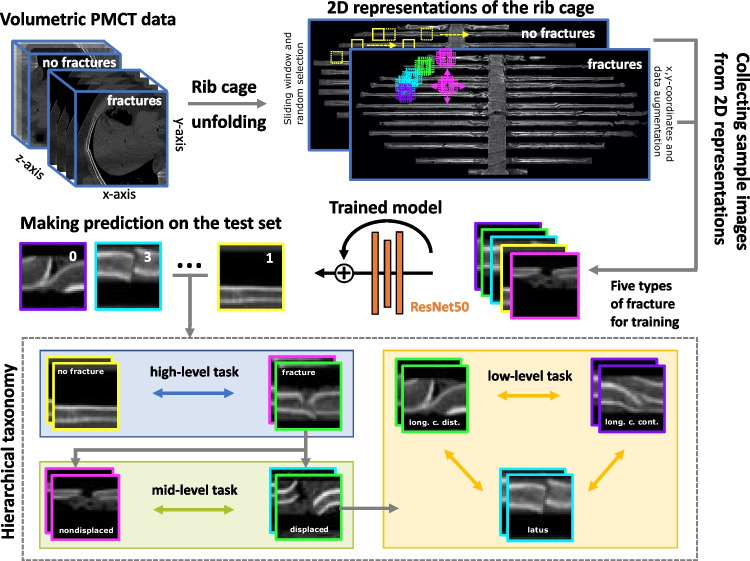
Workflow of the automated rib fracture classification pipeline. Each volumetric PMCT scan of the rib cage was transformed into a corresponding 2D representation. If the representation did not display any fracture (“no fracture”), we collected a series of sample images (each measuring $99\times 99$ pixels) using a sliding window. Then, we randomly drew from a subset of those samples. If the representation displayed rib fractures, we collected a sample at the exact position of the fracture with an additional set of 16 samples. The additional set was obtained using data augmentation by sliding the $99\times 99$-pixel window in each of the four cardinal directions in 10-pixel steps. The samples from the four fracture types and the “no fracture” samples were fed into a ResNet50 architecture for training and testing. We validated the performance of our model on three levels of hierarchical taxonomy: (1) a high-level task where the model distinguished between “fracture” and “no fracture,” (2) a mid-level task to assess how well the model could classify “nondisplaced” and “displaced” fractures, and (3) a low-level task to validate the performance of the model in classifying the three different types of displaced fractures “ad latus” (sideways), “ad longitudinem cum contractione” (in long axis compressed fracture), or “ad longitudinem cum distractione” (in long axis with gap between the fragments)

The rib fracture images were reconstructed from volumetric CT data using Syngo.via rib unfolding tool CT Bone Reading (Siemens Healthineers GmbH, Erlangen, Germany) with standard bone window setting (center 450, width 1500) (see Fig. 1 for more details). The tool used for this conversion was developed by Ringl et al. [19].

### Data mining

To extract data containing fractures, we used 270 images of unfolded rib cages with fractures. Two readers, one who was a medical student under supervision and one who was a board-certified forensic pathologist and radiologist, classified each fracture type as either “displaced” or “nondisplaced.” The “displaced” fractures were further divided into “ad latus” (sideways), “ad axim” (with angulation), “ad longitudinem cum contractione” (in long axis compressed fracture), and “ad longitudinem cum distractione” (in long axis with gap between the fragments). Due to the very small number of “ad axim” fractures, we excluded them from further analysis. First, we cropped the images to $500\times 1000$ pixels to eliminate the background and then upscaled the images to 300% of the original size with the INTER_AREA interpolation method from OpenCV, resulting in large images measuring $1500\times 3000.$ With this preprocessing step, we wanted to achieve an optimal size for dividing the image into sufficient image patches but still capturing all fractures. All fractures were marked using their respective *x*- and *y*-coordinates on the large image. For each large image containing one or more fractures, we applied data augmentation by shifting the sliding window from the centered *x*- and *y*-coordinates in all four cardinal directions (up, down, right, and left) in steps of 10 pixels. This resulted in a total of 16 additional samples next to the original sample (centered around the fracture). For each fracture, we then manually removed the sample images where the data augmentation resulted in a loss of information (e.g., the fracture was no longer visible). The sample curation led to 11,759 “displaced” (“ad latus” 1785, “longitudinem cum contractione” 6801, and “longitudinem cum distractione” 3173) and 18,462 “nondisplaced” samples, for a total of 30,251 “fracture” images.

To extract samples with the label “no fracture,” we used 231 images of unfolded rib cages without any fractures. As for the images with fractures, we applied the same preprocessing steps (cropping and resizing) to images without fractures. Employing a sliding window of size $99\times 99$ pixels and shifting it 25 pixels in each direction along both the $x$- and $y$-axes, we obtained 231,926 small images, each of which was $99\times 99$ pixels in size. From these images, we randomly selected 30,251 “no fracture” images, resulting in a balanced dataset of 60,472 samples in total.

### Training, validation, and testing

For our study, we used a Windows workstation (Windows 10, Nvidia GeForce GTX 1660 SUPER, 64 GB CPU RAM). We split our data into ~ 70% training and ~ 30% test data. Representations from the same fracture were kept together in each partition to prevent data leakage into the test set; thus, the partitions varied slightly in size. We then ran a 5-fold cross-validation on the training dataset with different hyperparameters. We selected the best hyperparameters (see Section “Model architecture and hyperparameters”) by assessing the epochs with the highest validation score (*F*_1_ score). Finally, we trained our model with the best selection of hyperparameters on the full training dataset and validated the trained model on the test set. We assessed three levels of hierarchical taxonomy (see Fig. 1 for more details):

1. Performance of the model on the balanced binary task when classifying “no fracture” and “fracture” and reported with the accuracy score (high-level task).
2. Performance of the model on the imbalanced binary task when classifying “displaced” and “nondisplaced” with the *F*_1_, precision, and recall scores (mid-level task).
3. Performance of the model on the imbalanced multiclass task with the displaced classes “ad latus,” “ad longitudinem cum contractione,” and “ad longitudinem cum distractione” with the *F*_1_, precision, and recall scores (low-level task).

Additionally, we defined two types of assessment:

1. Performance measurement on the fracture representations (referred to as “standard” assessment), as in simple image classification tasks.
2. Aggregation of the prediction values from multiple representations of the same fracture into a single prediction value. The aggregation procedure starts by running a custom-made function $Y$ on the predicted values. The function $Y$ is defined as

$$Y=\left\{\begin{array}{cc}0,& \mathrm{if}\;\sum\limits_{i=1}^{n}{\widehat{y}}_{i}=0\\ 1,& \mathrm{otherwise}\end{array}\right.$$

where the variable ${\widehat{y}}_{i}$ stands for the label value predicted by the model for the representation $i$. The variable ${\widehat{y}}_{i}$ can take any integer value from 0 to $c$, where $c$ represents the number of classes. Hence, the function $Y= 0$ if at least one of the representations $i$ was classified into the class 0 (classified as “no fracture”). Otherwise, the function $Y= 1$ if at least one of the representations $i$ was classified into a nonzero class (classified as “fracture”). Then, we used the maximum operator to determine the fracture type $k$ when $Y= 1$:

$$k= \underset{c}{\mathrm{max}}(\frac{1}{n}\sum_{i=1}^{n}{{logit}}_{i}^{c})$$

where the ${\mathrm{logit}}_{i}^{c}$ stands for the model output value for the class $c$ before entering the Softmax function. In other words, the aggregated prediction value corresponding to a single fracture is the type of fracture (class) that has the highest weight over all its representations. This would ensure us that we have detected a fracture even with the weakest signal. We referred to this type of assessment as “aggregated.”

### Model architecture and hyperparameters

We used the ResNet50 architecture [20] pretrained on the ImageNet database combined with two additional dense layers, each with 198 neurons, and with a dropout layer whose dropout rate was 0.5. Additionally, we included the EarlyStopping function to stop the training when the value of the validation loss function was minimal (patience = 15). We also used the ReduceLROnPlateau function to downscale the learning rate when the validation loss value was not improving (patience = 2) [21]. The batch size was set to 16, and we used the categorical cross-entropy loss function with the Adam optimizer. We first froze the layers of the pretrained network and trained on our data for several epochs (max = 100 epochs, depending on early stopping) with a learning rate of 0.0001. Then, we unfroze the layers and fine-tuned the network for another few epochs (max = 100 epochs, depending on early stopping) with a learning rate of 8e − 05.

## Results

**Fig. 2:**
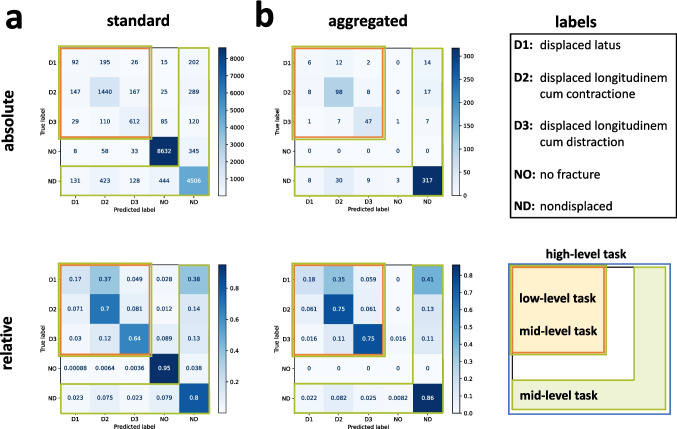
Confusion matrices. Top row: absolute values; bottom row: relative values. Blue, high-level task. Orange, mid-level task. Green, low-level task. **a** Confusion matrices for standard assessment (all data points) and **b** confusion matrices for aggregated assessment (aggregated to fractures as data points)

 We assessed the performance of our model in two different ways. First, we showed the metrics for the predictions on all representations in the test set (“standard” assessment). Second, we aggregated the predictions of all representations on the test set to the fracture level and reported the metrics (“aggregated” assessment). Figure 2 shows the confusion matrices for all classes in terms of absolute and relative values and for each of the assessments. Most of the confusions occurred within the fracture classes, while fewer occurred in the class “no fracture.” While “nondisplaced” was correctly predicted in 80–86% of cases (depending on the assessment), “ad latus” (sideways) was correctly predicted in only 17–18% of cases. The other two “displaced” subclasses, “ad longitudinem cum contractione” (in long axis compressed fracture) and “ad longitudinem cum distractione,” (in long axis with gap between the fragments) were correctly predicted in 70–75% and 64–75% of cases, respectively.

Table 1 gives an overview of the performance of our model. In the balanced binary classification task with the classes “no fracture” and “fracture,” our model achieved an accuracy score of 0.945 (0 worst score, 1 best score) on the “standard” assessment and an accuracy score of 0.993 on the “aggregated” assessment. When evaluating the models’ performance on the imbalanced binary task with the classes “displaced” and “nondisplaced,” we found an *F*_1_ score of 0.845, a precision score of 0.845, and a recall score of 0.846. When data were aggregated at the fracture level, the model achieved an *F*_1_ score of 0.856, a precision score of 0.857, and a recall score of 0.855. The third task was an imbalanced multiclass task of the different “displaced” classes “ad latus” (sideways), “ad longitudinem cum contractione” (in long axis compressed fracture), and “ad longitudinem cum distractione” (in long axis with gap between the fragments). There, we found an *F*_1_ score of 0.661, a precision score of 0.736, and a recall score of 0.603 for the “standard” assessment and an *F*_1_ score of 0.707, a precision score of 0.769, and a recall score of 0.662 for the “aggregated” assessment.

**Table 1:** Performance assessment overview. We assessed three different hierarchical taxonomy levels: (1) “fracture” vs. no “fracture,” (2) fractures separated into “displaced” and “nondisplaced,” and (3) displaced fractures separated into three subclasses. The three levels are assessed in two ways; “standard” (all images) and “aggregated” (e.g., all representations of a fracture aggregated into a single datapoint). For each case, we calculated the *F*_1_, precision, recall, and accuracy score, depending on whether the dataset is balanced (accuracy) or imbalanced (*F*_1_, recall, and precision)

	Standard				Aggregated			
	*F*_1_	Precision	Recall	Accuracy	*F*_1_	Precision	Recall	Accuracy
“Fracture” or “no fracture”	-	-	-	0.945	-	-	-	0.993
“Displaced” or “nondisplaced”	0.845	0.845	0.846	-	0.856	0.857	0.855	-
Displaced subclasses	0.661	0.736	0.603	-	0.707	0.769	0.662	-

## Discussion

The aim of this study was to train a deep learning model able to detect and classify different types of rib fractures using a two-dimensional representation of the rib cage reconstructed from three-dimensional PMCT images. By applying our model, we investigated two types of assessment (“standard” and “aggregated”) on three different hierarchical taxonomy levels (“fracture” versus “no fracture,” “displaced” versus “nondisplaced,” and “displaced subclasses”) with different scores. Our results show that the trained model can distinguish between “fracture” and “no fracture” samples to a large extent and with a high accuracy (94.5%). When data were aggregated at the fracture level, only three out of 591 fractures were classified as “no fracture.” The model also performed reliably in distinguishing “displaced” from “nondisplaced” fractures, although to a slightly lesser extent. When classifying “displaced” from “nondisplaced” fractures, we noted that the trained model performed slightly better in classifying “nondisplaced” than “displaced” fractures. This could be due to either the smaller sample size or the possibility that the features of “displaced” fractures were more difficult for the model to capture. Finally, the most difficult task was distinguishing “displaced” subclasses. In particular, the model performed worst for the subclass “ad latus” (sideways), which was often confused with “ad longitudinem cum contractione” (in long axis compressed fracture) or “nondisplaced.” The scores for the aggregated assessment were generally higher than those for the standard assessment, which reflects our choice of metric design. We defined a single correct fracture prediction from all possible representations as sufficient to qualify as a “fracture” and be classified accordingly.

As we mentioned in the introduction, three recent studies used deep learning techniques to automatically detect rib fractures either on CT scans or radiographs. These studies used different datasets which makes it difficult to compare their performance with our model. However, we went one step further by identifying four different subclasses of “displaced” fractures. We also developed a method to display the position of each fracture. If multiple fractures are present on the same CT scans, they are labeled separately (see Fig. 1).

## Conclusion

The analysis of two-dimensional representations of the rib cage instead of volumetric data already enables clinicians to make a quick and easy assessment for potential rib fractures. Building upon our previous work [22], we have shown how deep learning techniques can be used as an automation step to reliably locate and classify relevant fracture types on such large two-dimensional PMCT images and thus further simplify and support clinicians’ work.

## Key points

1. Our model achieved an accuracy score of 0.945 on a balanced binary classification task with the classes “no fracture” and “fracture.”
2. The *F*_1_ score on the imbalanced binary task with the classes “displaced” and “nondisplaced” reached 0.845.
3. Classifying “displaced” subclasses remains challenging, especially the subclass “ad latus.”

## Data Availability

The datasets analyzed during the current study are not publicly available due to data privacy. The code and the trained models are available on reasonable request.
